# Evaluating a Smart Textile Loneliness Monitoring System for Older People: Co-Design and Qualitative Focus Group Study

**DOI:** 10.2196/57622

**Published:** 2024-12-17

**Authors:** Freya Probst, Jessica Rees, Zayna Aslam, Nikitia Mexia, Erika Molteni, Faith Matcham, Michela Antonelli, Anthea Tinker, Yu Shi, Sebastien Ourselin, Wei Liu

**Affiliations:** 1 Department of Engineering King's College London London United Kingdom; 2 Department of Global Health and Social Medicine King's College London London United Kingdom; 3 Institute of Psychiatry, Psychology, and Neuroscience King's College London London United Kingdom; 4 School of Design University of Leeds Leeds United Kingdom; 5 School of Biomedical Engineering & Imaging Sciences King's College London London United Kingdom; 6 School of Psychology University of Sussex Sussex United Kingdom

**Keywords:** loneliness, smart textiles, wearable technology, health monitoring, older people, co-design, design requirement, mobile phone

## Abstract

**Background:**

Previous studies have explored how sensor technologies can assist in in the detection, recognition, and prevention of subjective loneliness. These studies have shown a correlation between physiological and behavioral sensor data and the experience of loneliness. However, little research has been conducted on the design requirements from the perspective of older people and stakeholders in technology development. The use of these technologies and infrastructural questions have been insufficiently addressed. Systems generally consist of sensors or software installed in smartphones or homes. However, no studies have attempted to use smart textiles, which are fabrics with integrated electronics.

**Objective:**

This study aims to understand the design requirements for a smart textile loneliness monitoring system from the perspectives of older people and stakeholders.

**Methods:**

We conducted co-design workshops with 5 users and 6 stakeholders to determine the design requirements for smart textile loneliness monitoring systems. We derived a preliminary product concept of the smart wearable and furniture system. Digital and physical models and a use case were evaluated in a focus group study with older people and stakeholders (n=7).

**Results:**

The results provided insights for designing systems that use smart textiles to monitor loneliness in older people and widen their use. The findings informed the general system, wearables and furniture, materials, sensor positioning, washing, sensor synchronization devices, charging, intervention, and installation and maintenance requirements. This study provided the first insight from a human-centered perspective into smart textile loneliness monitoring systems for older people.

**Conclusions:**

We recommend more research on the intervention that links to the monitored loneliness in a way that addresses different needs to ensure its usefulness and value to people. Future systems must also reflect on questions of identification of system users and the available infrastructure and life circumstances of people. We further found requirements that included user cooperation, compatibility with other worn medical devices, and long-term durability.

## Introduction

### Background

Loneliness is a common experience in later life and describes a subjective feeling, whereas social isolation is an objective state that is not necessarily associated with felt loneliness [1,2]. While people can be alone and lonely, they can also be lonely while cohabiting with their families [3]. Risk factors for loneliness and difficulties dealing with loneliness at an older age include physical health issues; lack of social resources, such as quality and frequency of contacts; lower educational attainment; and loss of a partner [3-6]. Loneliness can have health implications for individuals and economic impacts on the health care system. Loneliness has been shown to affect stress responses by increasing fibrinogen levels and diastolic blood pressure [5], risk of malnutrition [7], frailty [8], sleep deterioration [5], greater sensitivity toward self-rated health [2], depressive symptoms [9], and higher mortality risk [10].

Loneliness monitoring can help older people communicate with health care providers [11,12] and provide reassurance to family members. Another motivation is to assess loneliness objectively and understand its relation to health outcomes [11,13]. Previous systems used sensors at home, in corridors, and on doors and monitored phone and computer activities [11,12]. In this paper, we focus on the use of smart textiles in wearables and furniture for loneliness monitoring. Smart textiles have the potential to support a comfortable, accessible method of detection for physiological and behavioral data [14-16]. No previous systems designed to detect and systematically assess loneliness have used smart textiles for detection. Only 1 study detected the frequency of speech through a textile band; however, it did not evaluate whether this could be associated with the experience of loneliness [17].

### Lessons From Loneliness Monitoring Systems for Older People

Loneliness has been associated with depressive symptoms [18], sleep disturbance [19], sleep dysfunction, lower sleep quality, and differences in heart rate [20] and blood pressure [21]. Previous monitoring systems for loneliness also found associations with decreased daily phone use and incoming calls [13], time spent outside the home [11,12], the number of computer sessions [11], and speech characteristics [22]. Some of these symptoms could potentially be detected with smart textile sensors positioned on beds [23], embedded electrocardiogram sensors [24], sensors in insoles [25], or pressure sensors [26] on seat covers.

Design considerations for current loneliness monitoring systems concern the need for privacy, system unobtrusiveness [11,12], and durability (ie, battery efficiency) [27]. Having sensors installed intuitively on walls or devices was considered more comfortable and could be more easily remembered by older people than those on wearables [11]. There have been a few proposals for the application of a loneliness monitoring system. One proposal was to implement the technology in community settings to raise awareness for people who are on the verge of becoming lonely and to address the issue or share the data with physicians or nurses [11]. There is also a need to gain consent from older people to use the proposed technology [11]. Furthermore, a consideration is the question of how to serve lower-income and rural populations in accessing this technology [12]. No studies have presented in-depth findings on the needs and preferences of older people or stakeholders in loneliness monitoring systems using smart textiles. Thus, we drew on user studies and insights into design requirements from the literature on smart textile technologies.

### Lessons From Smart Textile Wearable Technologies for Older People

Compared to pervasive computing systems, smart textile systems are predicted to acquire denser data, be less obtrusive, and reduce the number of devices required to detect diverse symptoms simultaneously [28]. Greater privacy is also a reason for positioning sensors in insoles for monitoring falls compared to alternative video cameras [25]. Positioning of sensors in furniture, such as a mattress topper, has previously been considered less invasive than worn sensors [23]. Positioning sensors on wearables, such as socks, allows for continuous monitoring including at home [29]. Even in older patient groups, there was a high adherence to the continuous use of wearable sensors, helping continuous monitoring of potential health risks [30]. Other examples of textile sensors include Taekwondo suits (pants and jackets) [26], floor mats [31], waistbands [24], and head ornaments [32]. Previous applications have contributed to the development of strain, pressure [26], electrocardiogram textile sensors, and explored the possibility of sensors with combined functions [26]. There has been a proposal for “intelligent health agents” based on smart textiles, which can support monitoring and treatment through textile technologies, such as thermotherapy, electrical stimulation, or phototherapy [33].

The commonly mentioned requirements are comfort [34-36] and wearables being made of breathable [34,37], lightweight, and permeable materials [34]. Furthermore, smart textile applications would need to function during daily and sports activities [35], be discrete [36], be safe on the body, and resist bodily fluids [35]. Aesthetic properties, such as color, fit, and diverse choices, to adapt clothing to the cultural context of the wearer and the possibility of mass production of such design choices should be further considered for greater acceptance [35,38]. Moreover, smart textile wearables must adapt to different body measures, genders, postures, and movements [35]. Further requirements include greater accuracy, reliability, privacy, and safety of the transmitted information [38]. The requirements for the material can vary according to the application context, but they did involve resisting large deformations and strikes in sports [26] and resisting abrasions from moving a topper on a mattress [23].

Furthermore, washability is considered an essential requirement for smart textiles [34]. Current applications can range from approximately 5 washing cycles by hand [26] to 40 cycles at 25 °C by a machine [36]. This implies potential additional requirements for washing [39]. One proposed solution to protect the textile sensors was microelectronic systems that could “be detached from the clothing component before washing” [40]. However, this might also contradict being a user-friendly washing process [38]. Thus, it is recommended that smart textiles are machine washable, support known washing processes, and do not require complicated washing instructions [38]. Power sources are also vital as currently available power sources are often too bulky [41]. Hence, one requirement is reduced energy consumption as one way to use less bulky power sources and the option to use energy from motion and body heat [34,35,38]. Enough power should be provided to last throughout monitoring activities [35]. Another important consideration is the recyclability and potential biodegradability of smart textiles produced without using harmful materials [34,41].

We are an interdisciplinary research group consisting of experts in gerontology, psychology, design, sensor technology, and machine learning, aiming to develop a loneliness monitoring and communication system from a human-centered design perspective [42]. This study aims to understand the needs of older individuals and the expectations of stakeholders to inform the system design.

## Methods

### Overview

We obtained user and stakeholder feedback from workshops and focus groups to develop the design requirements and initial design concept of the smart textile loneliness monitoring system for older adults. The study design and topic guides were developed in collaboration with team members who are developing the textile sensors and explored symptoms of loneliness in previous interviews [40]. For instance, sensor development required an understanding of user expectations in the positioning of sensors, washing, and charging. In addition, our initial prototypes developed and presented during the studies were informed by insights from interviews on potential symptoms of loneliness.

On March 27, 2023, and April 12, 2023, we conducted 2 co-design workshops with people aged ≥65 years who had experienced loneliness (n=5). On June 7, 2023, and June 15, 2023, we conducted a second co-design workshop with stakeholders (n=7). Therefore, we derived a set of design requirements implemented in an initial service system scenario and a smart home wearable system prototype. In October 2023, the design requirements were evaluated in a focus group study that included older people and stakeholders (n=7).

### Participants

Participants for the workshop and focus group studies were contacted based on their previous expression of interest in the DELONELINESS project, as specified by Rees et al [4]. Invitations were sent via email newsletters within a housing network specialized for older people. We engaged individuals who had previously responded through a newsletter in a study involving older participants. Participants were also contacted through a study pool on wearable technologies from the Remote Assessment of Disease and Relapse-Major Depressive Disorder program focused on the passive monitoring of depressive disorders through smartphone data and wearables. Older participants were eligible for the workshops if they were aged ≥65 years, spoke fluent English, and experienced loneliness within the last 10 years since reaching the age of 65. Participants were ineligible if they had any cognitive impairment or dementia.

The severity of loneliness measured by the University of California, Los Angeles 3-item Loneliness Scale [43] has been associated with various health effects and restrictions. Higher and medium levels of loneliness were associated with a higher risk for frailty than lower levels of loneliness [8]. Another study showed that higher levels of loneliness were associated with greater mortality risk [9]. Higher levels of loneliness were also associated with people who might face functional restrictions, which have potential difficulties in engaging in activities [44]. Severe loneliness might have different requirements for a monitoring system and linked interventions. When participants were unable to attend on the day due to ill health, follow-up sessions were organized.

For both the workshop and focus group, stakeholders were recruited through the People in Research website by the National Institute for Health and Care Research to offer opportunities for public engagement and through the researchers’ professional networks. The eligibility criterion for stakeholders was being aged >18 years. We searched for family members of older people who had experienced loneliness, industry representatives, carers, social workers, social prescribers, housing providers, and charity workers.

Participant characteristics in the studies have been summarized in Table 1 and Textbox 1.

We chose a qualitative approach with the aim of gaining more in-depth insights into participant perspectives and needs, which serves well to inform the initial system design. Previous qualitative research on design requirements for technologies for susceptible people and with stakeholders have been based on sample sizes such as 12 patients and 4 stakeholders [45], 10 experts to develop and 4 to evaluate a system [46], or 15 stakeholders [47]. For a qualitative evaluation in user research, we followed the recommended participant number of 3 to 15 [48].

**Table 1:** Older participant demographics in the co-design and focus group study.

Characteristic		Study 1: co-design workshop (2 groups; n=5)	Study 2: focus group (2 groups; n=4)
Age (y), mean (SD; range)		74.6 (3.8; 70-83)	73.3 (5.4; 68-78)
**Sex, n (%)**			
	Female	4 (80)	2 (50)
	Male	1 (20)	2 (50)
Hughes Scale for Loneliness, mean (SD; range)		6.2 (1.7; 3-8)	5.8 (2.2; 3-9)
**Experience in using health monitoring technology, n (%)**			
	Yes	3 (60)	2 (50)
	No	2 (40)	2 (50)
**Living circumstances, n (%)**			
	Living alone	3 (60)	2 (50)
	Living with others	2 (40)	2 (50)

**Stakeholder characteristics in the co-design and focus group study.**

**Stakeholders in study 1: co-design workshop (2 groups; n=7)**

- Family member and private carer
- Representative for a housing network for older people
- Company founder for housing for older people
- Representative for a smart textile company
- Social prescriber and social worker
- Charity founder for loneliness
- Public contributor with non-English, international family background

**Stakeholders in study 2: focus group (1 group; n=3)**

- Family member and private carer
- Family member and care home manager
- Health and well-being coordinator (social housing)

### Procedure

#### Study 1: Co-Design With Older People

The first co-design sessions were held with older people to discuss their design preferences for the function and positioning of a textile monitoring system on the body and in the home environment. The session involved the activities mentioned subsequently, which were relevant to the overall system development. Topics include the positioning of sensors; what to detect; overall system components; and washing, charging, and service requirements.

Initially, participants were asked to brainstorm about behaviors indicative of loneliness and the strategies to detect them. They were also asked what objects they surrounded themselves with and in which situations they experienced loneliness. This served as a basis for further thought on how a system for monitoring loneliness could be positioned.

Participants received preprinted outlines of a human figure and a home environment in a task. They were asked to indicate through red and green markers where they would accept or avoid the positioning of sensors. The previously discussed and exemplary symptoms were added. Subsequently, each person shared their materials and presented them to the group. This first indicated how the system would be positioned and what should be detected. For the positioning, they were not presented with symptoms or preferred sensor positions from a technical perspective to avoid influencing them.

Afterward, a presentation by the researchers summarized specific questions relevant to the sensor development. Pictures and questions prompted the discussion on existing wearables (eg, t-shirts, underwear, and leggings), charging and washing requirements, and whether participants would accept detachable electronic components for wearables and furniture. They were also asked to comment on some proposed positionings for the system based on initially hypothesized symptoms and preferred sensor positionings as recommended by experts in our team.

#### Study 2: Co-Design With Stakeholders

**Figure 1:**
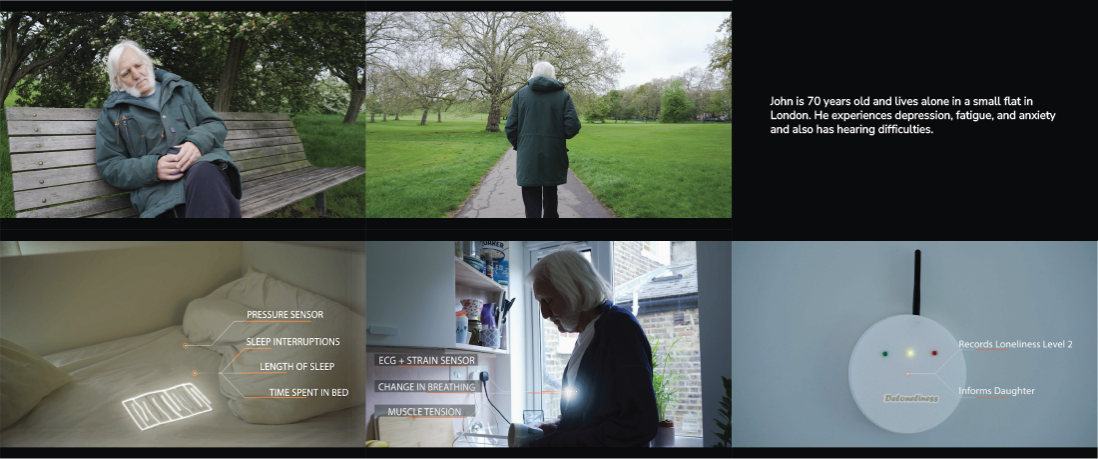
A short film about the main character, John, who experiences loneliness and uses the developed monitoring device. ECG: electrocardiogram.

The workshop with stakeholders was built on the previous co-design session with older adults, which helped inform and develop the first concept and use case and elicited further discussion (Figure 1). In addition, the stakeholder’s workshop aimed to explore the overall device implementation in the existing health care and personal environment. Therefore, the session involved additional activities mentioned subsequently.

First, participants were shown a video scenario (Figure 1). The video presents the case of John, an older man living with the loneliness monitoring system. It displays the locations where sensors are positioned, what symptoms are sensed, and what response is expected. The video shows that if a medium level of loneliness is detected, a family member will be informed, and they will initiate a call. A dedicated expert or social prescriber will be informed if severe loneliness is recognized. On the basis of the video, the stakeholder participants were provided materials to brainstorm about initial questions or concerns from their perspectives.

Afterward, stakeholders were presented with the human shape and home environment previously provided to older people to indicate how they suppose such a system should be positioned.

Finally, participants were asked to envision the implementation and installation of the system and the process while the researcher summarized comments in a sketch with preprinted elements.

#### Study 3: Focus Group Evaluation

**Figure 2:**
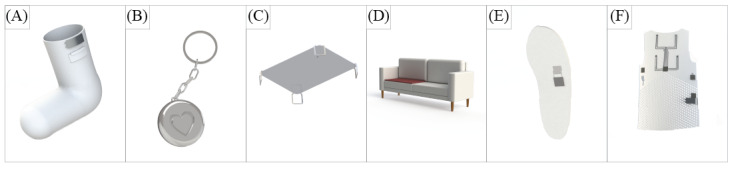
3D model of system components. From left to right: (A) a sock with a battery, (B) a sensor synchronization device shaped as a key charm, (C) a mattress cover, (D) a seating cover, (E) a shoe insole with an integrated pressure sensor, and (F) a vest that indicates the positioning of sensors and microchips.

A list of design requirements was developed based on the analysis of previous co-design sessions. These were implemented in digital and physical models (Figure 2) with materials recommended by the team members developing smart textile sensors. The focus group evaluated whether the proposed system design and requirement implementation were perceived as valuable and easy to use, which are predicting variables for older people’s use of new technologies [49].

The session was divided into 3 main parts. First, we presented the system’s physical components, showing its function and use process over time. Participants were asked to interrupt at any time to express their thoughts [50]. Afterward, open-ended questions followed, such as “What are your initial thoughts on the system?” “What are the benefits and drawbacks of how the system is positioned on the body and in the home?” and “Do you believe the system will be more or less useful to older people that were identified to experience loneliness?” In addition, a set of Likert-type questions (5-point items) was presented to get responses from everyone on the usefulness and expected ease of use (Textbox 2). The questions were inspired by the System Usability Scale [51] and measures for perceived usefulness [52]. Although participants had not yet tested the system’s usability, the rating was intended as the first evaluation step before the system was implemented and tested in the field.

In the second part, methods of washing and charging were presented. Washing would need to take place as efficiently as possible, as was determined by the previous co-design sessions. Participants were asked to rate the expected ease of washing, ease of remembrance, and comfort. Then, participants were asked open-ended questions, such as “What do you like or dislike about the way of washing proposed?” and “Would it be easy or difficult for an older person to maintain these forms of charging?”

**Figure 3:**
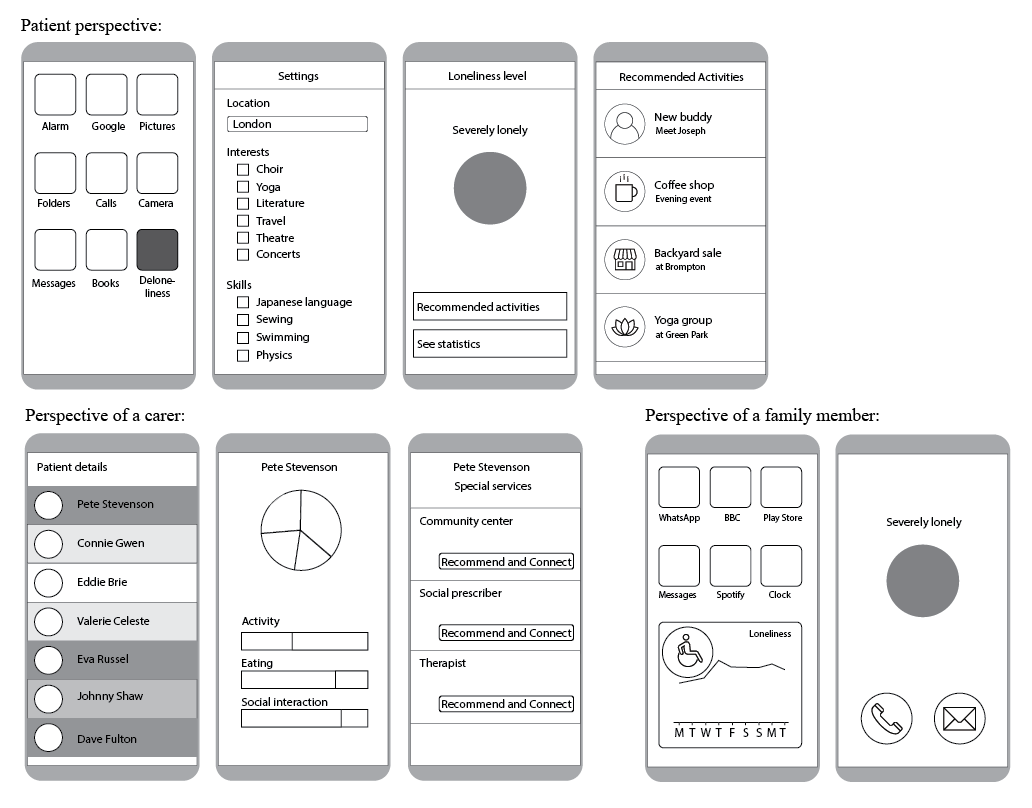
Proposed mobile app linked to the loneliness monitoring system.

Finally, participants were presented with 4 personas based on the previous interview data [4]. Participants were asked to comment on interventions that might be helpful to support the personas (Multimedia Appendix 1). Afterward, an initial proposal for a mobile app was shown and discussed (Figure 3).

**Rating questions asked in the focus group evaluation and 5-point scales.**

- The system can be used intuitively by older people: strongly disagree to strongly agree
- The system is useful to reduce loneliness for older people: strongly disagree to strongly agree
- How do you rate the comfort of the sensing (socks or mattress cover or seat cushion or vest or insoles)? not at all comfortable to very comfortable (1-5)
- How easily would older people remember to use the sensing (socks or mattress cover or seat cushion or vest or insoles)? not at all easily to very easily (1-5)
- The system is easy to wash for the older adult: strongly disagree to strongly agree (1-5)

### Analysis

The workshops and focus groups were transcribed by the first author and, in part, by a professional transcription service with the consent of the participants. Our analytical approach was an inductive qualitative content analysis [53]. Content analysis is a “technique for making replicable and valid inferences from texts...to the contexts of their use” [50]. A qualitative approach differs in coding, the determination of categories, and how researchers interpret data frequencies [54,55]. Categories are often developed inductively close to the dataset at hand and reading data in depth rather than by applying automatized analysis approaches based on predetermined codes [54,55]. According to the approach [53], we first applied open coding by reading the material, assigning codes, and developing categories with the help of NVivo (Lumivero) software. Then, the codes were classified and grouped into categories (eg, “accuracy in detection,” “economical inclusivity,” and “instructions for carers”). The categories developed were further grouped into superordinate categories (eg, “general system requirements,” “wearable requirements,” “installation and maintenance requirements,” “material requirements—wearable,” and “material requirements—furniture”). For part of the material, the coding was done independently by a second researcher. Differences in coding were resolved through discussion. This led to additional codes in a few categories. The categories were further presented to 2 coauthors. This led to the summary and further grouping of codes, such as soft and lightweight materials. Moreover, it led to renaming some categories, such as “machine washing” rather than “direct washing.” We considered frequencies and how often different participants mentioned a concept. The detailed quotes and frequencies in each category are enlisted in Multimedia Appendix 2. This served to structure the observed requirements. The frequency count is not intended to be statistically significant or generalizable but to better compare different requirements and their potential importance.

### Ethical Considerations

The study was approved by the research ethics committee at King’s College London (LRS/DP-22/23-34602) on January 16, 2023. Participants were provided with an information sheet and the consent form before the initiation of the study for their information and they were informed about being able to opt out at any time in the study and asked to sign the consent form on the day the study started. In all data processing, data were labelled with a unique non–personally identifiable participant identification number. This made the data pseudoanonymous. A password-protected file contained the name and contact details with an ID that is stored separately. Participants were not provided with any direct incentives to participate. However, travel and lunch costs were reimbursed.

## Results

### General System Requirements

The general system requirements addressed the overall system function or were mentioned concerning multiple aspects of the system.

#### Accuracy of Detection

Older people and stakeholders wished for the system’s effectiveness. Some older participants were skeptical about how a subjective feeling of loneliness could be associated with physical symptoms or behaviors:

A sensor’s not gonna pick up my feelings of emptiness.

They were also incredulous that sensors could accurately detect the intended symptoms:

I wonder if that would work with my dog. I talk to my dog all the time.

A couple of stakeholders suggested that sensors would need to be able to differentiate data from home visitors:

But when other people come to visit you or use your house or even if their care workers or something. Then they would they might access the toilet, the kitchen sink. They wouldn’t be in your bed though, you tend to have your own side of the bed don’t you. So, I think bed probably works well. But what about towels? I don’t know if in a towel would work.

The company representative and expert in smart textiles also noted the importance of data detection during the wearable’s movement.

#### Economic Inclusivity

Stakeholders with a background in charity, social work, or housing provision raised the question of how such a system could become affordable to more people. They recommended a tiered system:

Being a tiered service, a basic service, open to all. And if I can afford it and can afford to pay a subscription maybe a better-quality service or a wider range of monitoring.

People could choose from a basic or more elaborate sensor system and intervention for loneliness based on their financial possibilities and individual needs.

#### Cultural Inclusivity

Some stakeholders questioned the compatibility of such a system for different cultures. For instance, some cultures might prefer seats on the floor rather than a traditional settee:

Some people might not use settees but sit on the floor.

The monitoring system and linked interventions would also need to be delivered in multiple languages or visual symbols that are universally understood:

Different cultures respond to different phrases.

#### Flexibility to Change

An expert on housing provision to older adults mentioned the importance of system flexibility concerning future technological developments:

Builders and housing developers want something flexible. Because if you look at things like the existing, the old-fashioned emergency call systems, a lot of providers had difficulties retrofitting.

Previous emergency call systems had proved challenging to replace in care housing. Thus, systems need to avoid becoming integrated into the home infrastructure.

#### Comfort

Comfort was mentioned by some older participants in workshops, describing it as a crucial element of wearable acceptability:

But then surely we are back to the Velcro thing, aren’t we, which we discussed, and we don’t really like because it is stiff and uncomfortable.

It was a key argument for where they wished the sensor system to become positioned on the body. Older adults expressed concerns regarding wearables with detachable components, with 1 participant describing Velcro as “stiff” and “uncomfortable.” The mattress cover and seating cushion were rated most comfortable (mean 4.5, SD 0.56). The vest was perceived as less comfortable (mean 3, SD 0.71). One participant felt he would be unwilling to wear a vest but would accept a watch:

I could see being prepared to wear a watch.

It also seemed related to the fact that participants felt concerned about the comfort of such a vest:

It might go underneath of their own vest or if it can go on top of their vest, there are two sides there. Their own vest is comfortable, and they are used to it so the other one could be on top of it, if it still works.

#### Environmental Consciousness

Concerns about the environmental impact of the technology were expressed by 3 of the older participants. Environmental consciousness affected peoples’ charging and material preferences. Moreover, a stakeholder with experience in providing smart textile applications emphasized the importance of enabling the exchange of batteries at the end of their life span to avoid the wastefulness of the overall technology. It needs to be outweighed whether people want easier washing or longer durability of the components. Participants disagreed that it would be acceptable to dispose of a vest after 3 months of use (mean 2, SD 1.41). In addition, participants disagreed with disposing of the detachable sensors after 3 months of use (mean 2, SD 1.50), indicating environmental and economic consciousness.

#### Cooperation With Other People

The social worker reported cases where people liked manipulating activity monitoring and supporting systems:

It’s actually critical, is accuracy. I think you mentioned earlier that, you know, if people are given a walking stick with G.P.S. monitoring, they can give the walking stick to somebody else and wander off.

A family member reported that their relative misused a sensing doormat to call staff more frequently, so it had to be unplugged:

My mother is actually in a residential home. She started off with a mattress cover and a floor mat, but when she gets annoyed at the staff, she consciously hits it to make sure that the staff are required to come and see her. And they have given up with using them because she does it so often and they have to respond to her.

Thus, the monitoring system’s functioning will need people to cooperate, and developers need to expect that some people are willing to trick or use a system in unintended ways.

#### Aesthetics

In response to the presented digital and physical system concepts, focus group participants felt that aesthetics and a gentle appearance were missing. The system components were found to be too sterile and cold in appearance:

It does remind me of the original cars in America when everything was black, black, or black and this feels like it’s surgical. It doesn’t have a pretty pattern for example.

The indication of loneliness in graphics or visual data also needs to convey information more sensitively. A red color or light on a display or device might indicate a lower level of physical and mental well-being or a need to charge the device. However, this needs to be designed in a more sensitive and friendly manner. According to an older participant, red colors should be avoided and instead, more friendly, soothing colors should be used:

That one goes red, they wouldn’t like that. Now they know that I feel lonely, you know, because there is a stigma attached as well, so sweet colors, you know the meaning of them, but you don’t want to scare them.

### Requirements for Wearables and Furniture

#### Compatibility With Existing Clothing and Furniture

A few stakeholders asked how a wearable would integrate with people’s existing wardrobe and how it would match the broad range of clothing styles. They felt that everyone had their preferences and style in clothing. Two older women also expressed how certain wearables, such as socks, would not suit dresses or tighter shoes, and a wearable would need to be compatible with different outfits and match one’s wardrobe:

If you are wearing skirt or dress you are not going to want to wear socks, aren’t you?

Many participants preferred the system’s attachability to existing furniture (eg, cushions). Due to sentimental and financial reasons, older adults expressed that it was unlikely that people would be willing to exchange their existing furnishing:

I can’t see people being able to afford to have new furniture.

#### Seasonal, Intuitive, and Unisex

Participants from both interviews and workshops noted that a wearable must function in different seasons:

On a summer’s day you wouldn’t have it on the sleeve.

Interview participants frequently mentioned underwear (ie, brassiere and pants) as an item of clothing that is close to the skin and intuitively worn daily:

I think the picture with this attached to your bra makes the most sense to me, because it is something you automatically put on and you wear under your clothing.

Older adults in workshops expressed the importance of integrating a wearable sensor into clothing that would be habitually worn to avoid additional burdens. While most women in the first co-design session agreed to have sensors positioned on bras, they felt it would not be adequate for men:

It would be a problem for men, presumably men, I mean we are all female.

Thus, unisex options for the wearables would need to be available, such as vests and socks.

#### Discretion

The device’s conspicuous design or positioning could risk drawing unwanted attention. A few workshop participants expressed concern that a conspicuous design or positioning (eg, wrist-worn wearable) could also feel stigmatizing or become the subject of conversation:

I think people would ask you what it was. I think you would draw attention to it if it was on your wrist.

The loneliness sensing system would need to be presented inconspicuously.

#### No Detachable Components on Wearables

Detachable sensor components were proposed to support the sensor's durability over washing cycles. The detachability of sensors could also enhance the recycling of the sensors on the garment and reduce waste. Multiple participants argued against attachments to apparel and clothing due to comfort, cleanability, and the risk of breaking the sensors. There was a risk that Velcro would feel “bulky,” and detachable components would still need to be cleaned, as they were close to the skin. Furthermore, people could easily forget to remove the detachable components and accidentally break the device:

People will forget to remove stuff...Things will break because of that.

### Material Requirements

#### Lightweight and Soft Materials

Older adults from workshops mentioned the need for lightweight materials to increase the discretion of the device (“You want to make it seamless”) and the need for greater comfort. Participants recommended soft materials or materials that are not rough. It is important to select a preferred and comfortable material, particularly for textiles worn close to the skin continuously.

#### Natural and Antiallergic Materials

Most participants preferred natural materials for comfort and mentioned the likelihood of allergic reactions to synthetic fabrics:

And cotton I suppose because some people are allergic to, if I wear something artificial over a certain percentage, I get rashes all over the place.

#### Nonmagnetic Materials

A private carer mentioned the potential interference of sensors with pacemakers worn to regulate one’s heart rhythm. Thus, all components embedded in clothing will need to be noninterfering.

#### Wrinkle-Free Materials

One older participant also wished for a material that does not wrinkle, to avoid ironing it. Otherwise, it would need to be a material that resists ironing:

Because some shirts nowadays, you know, you don’t really need to iron them.

### Positioning Requirements

#### Privacy

Privacy was a key determinant in how participants positioned the sensing textiles on the body and in the home. Most participants mentioned they would not position any wearables closer to the lower torso region, excluding shorter pants or underwear in that area. Moreover, some individuals found the positioning of sensors in the bed or bedroom unfitting due to privacy issues. Conversely, 4 participants found positioning in the bed and bedroom reasonable and acceptable. Therefore, privacy needs vary from person to person.

#### Use in Daily Activities

The preferred positioning of smart textile wearables on the body and furniture also depends on people’s daily activities. Some participants felt that a sensor positioning on longer sleeves, socks, or in the bathroom would pose a risk of getting the sensors wet. This would lead to a more frequent need to wash the wearable or home-based device. Interview participants also highlighted the importance of the wearable being discreet and unnoticeable, with specific reference to its use, and not being inconvenient or impacting daily activities. A wearable must be integrated to appear subtle, hidden, imperceptible, and inconspicuous.

### Washing Requirements

#### Washability of All System Parts

Older adults in workshops discussed how any textile would need to be regularly washed as they would be in close contact with the skin. In terms of furniture, covers must be regularly washable, for instance, in cases of incontinence or spills, whereas clothing wearables, specifically underwear, would need to be washed daily. Workshop participants also discussed how any detachable components close to the skin would need to be clean and washable. Therefore, the detachable components would take more effort to clean than when directly printed on the wearable, but they could be treated more sensitively.

#### Machine Washing

The device or wearable should be machine washable as not everyone might like to do a separate washing, as mentioned by few individuals. More individuals preferred a wearable that allows a washing without having to remove any detachable components beforehand (eg, batteries and sensors). This also means that the sensors must resist stronger kinds of detergent and need printed batteries. Participants were neutral (mean 3, SD 1.07) regarding whether they found the system easy to wash. Some were happier to apply a special washing cycle:

I like doing hand washing. I hand wash a couple of times a week.

Others were more critical and unwilling to introduce a separate gentle washing cycle:

I only use one cycle on my washing machine and it’s going to destroy this. I use the hand washing cycle about once every five months.

#### Life Span of 2 Years

A wearable would have an expected life span; however, it was difficult for participants to determine an exact period. The industry representative suggested 2 years after an estimation of yearly washing cycles. This was deemed an acceptable life span for consumers.

### Requirements for the Sensor Synchronization Device

#### Memory Support and Prevention of Loss

The sensor synchronization device is a technical component that helps collect and transmit sensor data outside the home. This could be integrated into a phone or kept as a separate device that an older person can carry. The device should be easily remembered when going outside:

It is much less likely that they forget about it if it is attached to their key or to their trousers.

The industry expert on smart wearable textile applications found a synchronizing device could be easily forgotten and this should be fixed so that people automatically carry outside with them. Alternatively, they could be reminded by a light or noise. One older participant felt a device of a smaller size could be more easily lost. In the evaluation, the mattress cover was rated highest for being easily remembered (mean 4, SD 0). The insoles and socks were considered less easy to remember for an older person (mean 3, SD 0.71). Participants were neutral (mean 2.8, SD 0.75) when asked whether the system is easy to use, also due to potentially forgetting to use it: people “might forget or not bother using it.”

### Charging Requirements

#### Little Cognitive Strain and Notifications

A couple of stakeholders also mentioned that older people might forget how to change batteries. Carers would potentially be supportive of charging and exchanging batteries for the devices. Notifications were a recommended feature by the industry expert. A display, light, or notification sound could help inform older adults and carers about the charging status. On average, participants in the focus groups disagreed on whether it would be acceptable to remove a battery before washing (mean 2, SD 1.06). Written comments included “too fiddly,” “not practical at all,” and “dexterity.” This is particularly relevant for batteries attached to the vest or socks, due to more frequent washing. When questioned about their thoughts regarding an inductive charging station, participants wished for a dedicated area to place all device components to be loaded:

If there was a single place that you could throw the whole lot and they be charged in the morning.

#### Fewer Plugs

The industry expert who had previous experience working with older people with disabilities mentioned that there can be few plugs in apartments. Therefore, the system should not require too many plugs:

We have a project with older people who also have learning disabilities. People don’t necessarily have a lot of power, lot of plugs.

#### Accessibility

Some charging processes can require the agility of the hands. While many found the idea of smaller batteries preferable, participants were concerned about the size and process of replacing batteries:

I would have a comment about removable batteries and everything because I know of at least two people who have problems with hearing aids putting batteries in, and it depends how like fiddly it is. For people who don’t have the dexterity.

Older people could have arthritis and might find certain charging processes difficult. Simple charging methods that require less precision, such as inductive charging, might be an appropriate solution.

### Requirements for the Linked Intervention

#### Providing a Linked Intervention

Participants were neutral on whether they considered the system useful to help address loneliness (mean 2.7, SD 1.25). Many older participants found it difficult to comprehend the value of the monitoring system, as they did not see how it could immediately help them address their loneliness. A notification about their loneliness was not perceived as valuable. Few older participants determined that they were already aware of when they felt alone. One participant also did not have any children or family members to be contacted. Thus, determining how the interventions are valuable to different individuals is important to their perceived value. This finding was also reflected in the focus group evaluation, where it became apparent that people were pressing on what happened due to the detection of loneliness. One participant expressed as follows:

I am not sure what help I will be for this research. If I know I am isolated or lonely, why do I need that? I don’t need that to tell me.

It is useful to use the system as an older person that experiences loneliness. Yes, it depends on what you do about it... The system is useful to reduce loneliness for older people, not in itself. It depends on what is done because you are just monitoring.

#### Personalization

Multiple stakeholders mentioned the importance of personalization concerning the linked intervention. A personalized intervention could better appeal to people who might feel discouraged from engaging in activities:

All of your interests. Something that really interests you and you will make the effort.

#### Avoiding Burden on Family Members

The monitoring system could communicate data to family members. However, a few older participants expressed concern about how such a system could burden their family members:

I don’t want it to be family because I think I mean I’ve got this memory of my grandmother guilt tripping people all the time. And I think this would be a very formal way of guilt tripping. And you know, they’ve got their own lives.

#### Infrastructure

Some stakeholders were concerned about not only the functioning of the technology but also the infrastructure requirements:

You felt you were having a heart attack and you phoned nine, nine, nine, it still might take six hours. So, I suppose that’s not that this doesn’t change that, does it. That’s an extra thing.

In addition, from the perspective of a family member, it was also noted that older people might be in a carer position themselves, which restricts their time and resources to take action to address their loneliness. One participant recalled previous experiences with dedicated staff for loneliness and long waiting times for being contacted. Infrastructural requirements are particularly relevant for interventions that respond to detected loneliness.

#### Motivation

One difficulty addressed by a few stakeholders was how to help with “chronic” or almost paralyzing loneliness. In such cases, older people might struggle to find motivation or see purpose in acting to change their situation:

For people who are lonely or who are isolated, it’s generalized again but they are not necessarily, they are in that situation because they potentially aren’t the type of person...or there aren’t the opportunities to go and do something. Actually, stepping through the door of some kind of group activity for some people they just wouldn’t do it.

### Installation and Maintenance Requirements

#### Identification

One of the first questions to be addressed is identifying people most in need of receiving such a system:

You need to know whether they’re likely to be lonely in the first place.

The monitoring system could become privately acquired, but it could also become part of housing schemes. Stakeholders discussed that identification should occur through individuals in regular closer contact with the older person, ranging from medical staff to people in the community and delivery service staff, as they can sometimes identify changes over time or be the only existent touchpoint. The social worker drew attention to the potential stigmatization connected to revealing one’s feelings of loneliness, and it might be preferable to make such a system the norm and offer it to everyone as part of the housing scheme.

#### Consent

Consent needs to be acquired before installation. Consent should confirm that people are willing to have such a system installed in their homes, and it should indicate who can access the data collected. Further considerations need to be made in case someone cannot make an informed decision for themselves:

The person whose data people are accessing, they need to give people permission, and they might not want to share certain things with the carer, they might not want to share with the family.

#### Easy Installation, Instructions for Carers, and Continuous Support Provision

The system needs to be easily installable, as recommended by an expert in textile wearables. It should ideally function readily when it arrives (“plug and play”). Carers will likely be involved in helping to charge the system or washing the textile wearables and furniture covers. Therefore, clear instructions need to be provided at the older person’s home. The instructions should be visibly placed as one needs to expect a regular change in carers, who will need to be directed. According to the industry expert, a technology provider would also need to have a customer service facility that can be called to provide help in case people have technical difficulties.

## Discussion

### Principal Findings

This research aimed to understand user and stakeholder requirements for a smart textile loneliness monitoring system. Overall, a key finding is the participants’ concern regarding what applications and interventions become linked to the sensor monitoring system for loneliness. Previous work found that older adults’ acceptance of smart textile technology and smart home devices depends strongly on perceived usefulness [56-58]. Whether the monitoring system was perceived as useful often depended on what actions would be taken in response to the monitoring. Previous rationales for developing loneliness monitoring provided in the literature were the importance of loneliness as a health concern [11,59]; the identification of loneliness, as directly communicating it might be associated with stigma [11,12,22]; potential bias in self-reporting and the nonobjective reporting of one’s state, also due to cognitive decline, to help prevent loneliness early on [11]; and research purposes to better understand the relationship between loneliness and health outcomes [11,13]. While questioning older participants and stakeholders, we found that participants perceived particular applicability in dementia, particularly as family members who wished to be notified of the status of loneliness of their loved ones. This might require future research to better involve such susceptible populations in the development of loneliness monitoring systems for accuracy. In contrast, it raises issues of consent and the state at which it would be achievable. Other potential applications considered by participants were buddy systems, being part of a group to improve technology development for older people to provide agency, and an artificial intelligence–recommendation system.

A proposition has also been made to potentially detect changes in individuals earlier on, in terms of loneliness, than is currently possible [13]. These suggestions elicit questions about available infrastructure and about an efficient distribution of the system. In case a loneliness monitoring system is intended warn people of loneliness in its early states [13], it could require a wider distribution than what might be affordable. A suggestion was to involve people who are in regular contact with potentially affected people to support a more targeted distribution.

Our findings showed that some older adults felt that the monitoring process did not solve their loneliness directly. Some also thought that they would not need a system to tell them when they are lonely.

Other propositions are informing family members or caregivers [12], overcoming stigma to receive help where necessary, and assessing current risks for greater mental health consequences [12]. Some declined the idea of contacting their family members in response to detected loneliness due to fears of burdening them. Stakeholders also noted that time constraints by health care professionals or social work professionals would not change via the introduction of a monitoring system. In contrast, 2 family members wished they had such a system to inform them about the well-being of susceptible family members (eg, those with mild dementia) across distance.

Similar to previous literature, participants expressed a preference for natural, soft, and lightweight materials useful in daily activities [34,37]. They also expressed a need for comfort [34-36] and discretion [36]. We found that furniture-embedded smart textiles (on a mattress or sofa) were rated more positively regarding comfort and ease of remembrance than smart apparel (eg, a smart vest), which has been predicted in previous literature on loneliness monitoring systems [11,23]. This was not only due to the individual preferences in clothing (whether one was used to wearing a vest or a watch) but also due to the greater potential effort in washing and charging. Therefore, it should be possible to consider individual preferences in the choice of sensor devices for monitoring [58]. In addition to the cultural sensitivity for clothing and wearable styles [35,38], we observed the same considerations in the context of textiles on furniture. In our study, we observed that colors that could elicit negative emotions should also be avoided, particularly for mental health applications. This aligns with findings from previously developed websites, robots, and devices to address the loneliness of older people [60-63]. Many vests and smart textiles developed for older people do have a plain design [64]; however, there have been exceptions that experimented with more friendly motifs of cats and flowers on smart textiles for patients with dementia [65]. Participants required the current system to function along with their existing furniture and clothing as they were unlikely to be replaced. Embedded electronics and their positioning must be compatible with other wearable health care devices, such as pacemakers or stoma bags. Regarding considerations of reliability and accuracy [37,38], participants also noted the possibilities of manipulation by them and the potential impact through people’s movement.

Washing and charging procedures are key concerns for the usability of the proposed technology. Current washing cycles [26,36] are not sufficient for user requirements, as participants did not agree with a life span of approximately 3 months. Most people did not accept special washing requirements, such as hand washing, or the use of a special washing cycle [39]. The proposed solution of detachable components [38,40,65], which could protect electronics from being washed as part of the overall textile, appeared more reasonable for furniture but less applicable for wearables that must be washed often. Participants emphasized the importance of washability of all parts (in particular, the materials close to the skin). Smart textile wearables and furniture covers are required to be washed frequently, due to the risk of spilling liquids at an older age or the possibility of incontinence. For maintenance, there will also need to be notifications and instructions for charging and washing and the requirement for fewer plugs, which is supported by previous findings from technologies addressing loneliness for older people. While current charging options are developed for smart textiles to become less obtrusive, they need to consider the potential limited dexterity of older people to handle them while washing or charging.

### Strengths and Limitations

There has been little understanding of the needs of stakeholders and older people for the development of smart textile loneliness monitoring systems. Our insights were based on people envisioning the system by presenting a prototype and use case. Therefore, it was not possible to determine the proposed system’s actual usability. In the future, we would like to undertake system testing in the field.

### Conclusions

This study presented key design requirements for a loneliness monitoring system embedded in smart textiles in wearables and smart home furniture from the perspectives of older people and stakeholders, touching on questions of usefulness and usability. Sensors on smart home furniture seem to face fewer difficulties in terms of individual habits, washing, and charging than applications that are wearable. Overall, the linked intervention in response to monitored loneliness remains a question that needs more in-depth understanding, including what individuals perceive as most valuable in connection with such a sensing system. We found additional aspects in the system design that have not found much consideration in previous work, such as the need for user cooperation, compatibility with other worn medical devices, user expectations regarding life span and washing requirements, questions about identifying the recipients of the system, and initial insights into possible linked interventions and purposes of loneliness monitoring systems.

## Appendix

Multimedia Appendix 1 Personas used to inspire the discussion on interventions.

Multimedia Appendix 2 Categories and frequencies (each participant is only counted once in each category) of quotes for the co-design and focus group study.
