# Supplementary material for: Evaluating a Smart Textile Loneliness Monitoring System for Older People: Co-Design and Qualitative Focus Group Study
Source: JMIR Aging. 2024 Dec 17;7:e57622. doi: 10.2196/57622 (PMC11688591; doi:10.2196/57622)
Supplement: Multimedia Appendix 1 [file aging_v7i1e57622_app1.docx]

**Multimedia Appendix 1**

**Table 2.** Personas used to inspire the discussion on interventions.

| Persona 1 | Aubrey is 70 years old. She likes the feeling of keeping healthy through her activities. She regularly uses technology such as Fitbit, Google Nest, and apps to monitor her activity levels, sleep, and other health-related factors. She does her exercise regularly every day. Having health data about her loneliness would be an efficient way to communicate with the GP and a good motivation for self-improvement. She hopes to receive some recommendations in response to the healthcare data collected. She does not want the system to inform family or friends and avoid being a burden to them. |
| --- | --- |
| Persona 2 | Jameson is 85 years old. As he aged, he suffered more from increased memory loss (health concern) and became more dependent on family and carers. He saw the loneliness detection system as a way to inform family members when he could not communicate his situation well by himself any longer. He hopes his family will contact him in case he feels alone. |
| Persona 3 | Theo is 70 and severely lonely. He has no children and feels he has no closer friends or contacts he can talk to when feeling lonely. He would be unsure who to nominate to be alerted by a loneliness monitoring system as he does not have contacts close enough to inform. He would like to have someone to call or speak to when feeling lonely, or someone to come over and visit him. He does not want an intervention that sends an alert but to have a real conversation. |
| Persona 4 | Ari is 75 and feels unfamiliar with most new technologies. She does not have Wi-Fi or a smartphone and has had difficulties downloading apps. She would appreciate an alert for others to know she has not been out for a while, and whether she is okay. She hopes a monitoring system will help her with suggestions for reducing loneliness. She would also like to be connected to something like a buddy system. She would wish for a user-friendly guide for the system. |
