# Supplementary material for: Evaluating a Smart Textile Loneliness Monitoring System for Older People: Co-Design and Qualitative Focus Group Study
Source: JMIR Aging. 2024 Dec 17;7:e57622. doi: 10.2196/57622 (PMC11688591; doi:10.2196/57622)
Supplement: Multimedia Appendix 2 [file aging_v7i1e57622_app2.docx]

**Multimedia Appendix 2**

**Table 3.** Categories and frequencies (each participant is only counted once in each category) of quotes for the co-design and focus group study

| Category | | Co-Design | Focus Group | Count |
| --- | --- | --- | --- | --- |
| General System Requirements | Accuracy in Detection | *“It's actually critical, is accuracy. I think you mentioned earlier that, you know, if people are given a walking stick with G.P.S. monitoring, they can give the walking stick to somebody else and wander off.” (1. Stakeholder)*  *“Is it monitoring multiple health conditions. Your heart rate's going faster. Is it because you're panicking or are you feeling down or is it because you are having a heart attack?” (2. Stakeholder)*  *“But when other people come to visit you or use your house or even if their care workers or something. Then they might access the toilet, the kitchen sink. They wouldn’t be in your bed though; you tend to have your own side of the bed don’t you. So, I think bed probably works well. But what about towels? I don't know if in a towel would work.” (2. Stakeholder)*  *“It depends how good the signals going to be and how much. Because when you go to the doctors, they put electrodes that stick to your body, right? So, they are attached to the body, so no matter how much you move, the sensor is gonna stay where it is. With clothes the fabric moves. You put your arm like that to fabric is going to move you. You move like that, the fabric is gonna move. You breathe. The fabric is gonna move, so that's why I'm thinking probably here. You're gonna have the least amount of movement when you know, moving your arms or whatever. “(3. Stakeholder)*  *“A sensor doesn't seem like I mean it's like a sensor's not gonna pick up my feelings of emptiness. A sensor is not gonna pick up that. But maybe your question is maybe you're wanting to find out whether actually it does.” (1. Participant)*  *“Whether actually there are ways that this can be physiologically concretely identified.” (1. Participant)* | *“I wonder if that would work with my dog. I talk to my dog all the time.” (1. Participant)*  *“So how many times you open your main door and how many times you open the fridge, okay. It is a bit off as a measure because people keep their cider in the fridge or other things, you know they might have drinks, they might look it up. They keep forgetting at a certain age so they might look at it just to see what is in the fridge rather than eating.” (2. Participant)*  *“It could be because they keep themselves company by saying, well done [RES] you got up this morning as well, and you don’t know whether they are talking to someone or to themselves.” (2. Participant)*  *“My mother was spending lots of time in her bedroom watching TV, so that was sensing well she stood possibly at the curtains but what would it tell you?” (1. Stakeholder)*  *“A typical person with dementia is going to open and close the fridge door many times in the day, how will you tell, it was seventeen today, thirteen yesterday?” (1. Stakeholder)* | 7 |
|  | Economical Inclusivity | *“Being a tiered service, a basic service, open to all. And if I can afford it and can afford to pay a subscription maybe a better-quality service or a wider range of monitoring” (1. Stakeholder)*  *“I just keep thinking whether there’s like a low level that’s cheap to get on board with and then on a more complex level if you want sophisticated stuff. So, some of it is down to how much you can afford and some of it is down to what you want to afford as well.” (2. Stakeholder)*  *“Unfortunately, obviously looking at the cost of cotton nowadays, it's too much. And I think the cost-of-living crisis is something we should always factor in” (3. Stakeholder)* | *“I mean as a one-off cost it’s relatively reasonable, not for everybody, I guess. I know lots of people, certainly it’s generalized but, you know, potentially in social housing people are on housing benefits they may not be able to have, especially two hundred pounds, and I think that will be the difference. It’s how much it, it’s what is putting in and when and what it is leading to.” (1. Stakeholder)*  *“Can you only buy the whole kit; you can’t buy individual parts? So, you couldn’t decide that actually you didn’t want the insole and the vest, but you did want the other things?... Because it will bring the cost down as well so you could maybe have an entry level and then also the additional things if you wanted them.” (2. Stakeholder)* | 5 |
|  | Cultural Inclusivity | *“Some people might not use settees but sit on the floor”. (1. Stakeholder)*  *“We could introduce it in different languages.” (2. Stakeholder)*  *“Different cultures respond to different phrases”. (3. Stakeholder)* |  | 3 |
|  | Flexibility to Change | *“Builders and housing developers want something flexible. Because if you look at things like the existing, the old-fashioned emergency call systems, a lot of providers had difficulties retrofitting”. (1. Stakeholder)* |  | 1 |
|  | Comfort | *“Because half the Velcro, one side of the Velcro has got to be attached to a garment. [ Are you talking about the comfort here then or is it easier washing?] It’s both.” (1. Participant)*  *[Is there anywhere on the body where you absolutely don't want it.] “Well, I suppose the lower torso. Don’t really care apart from that. [Can you briefly explain why so we know your reasons. Just so we understand]. Privacy areas and comfort I suppose.” (2. Participant)*  *“But then surely we are back to the Velcro thing, aren’t we, which we discussed, and we don’t really like because it is stiff and uncomfortable.” (2. Participant)*  *“Well, I mean it would depend what you came up with in terms of what it would feel like because obviously if it was uncomfortable, I wouldn't want to wear it. But I mean potentially, I think I would be prepared to try it.” (3. Participant)*  *“Did you ask us about the diaphragm? You didn’t particularly, did you?... I don’t think I would like to have something around my diaphragm. I think I would, potentially. If it were just a band around there. I’m imagining it might sometimes affect my breathing, but I don’t know It would depend how comfortable it was, you know if it was sort of really. It would depend on whether it was tight or not, if it was at all constricting, then I definitely wouldn’t like it” (3. Participant)*  *I'm not sure really. But I, as I said, my back turns I do get patches of eczema, so I wouldn't want it there. And I don't know. I just wouldn't feel comfortable anywhere else. (4. Participant)*  *For me, can I just be really just just straight about it. I don't really care for me, I mean, providing it is not uncomfortable, I don't mind. I mean, it's about whether it's effective. (5 Participant)*  *That's the thing with wearables, you want to make it as seamless and lightweight and comfortable. (1. Stakeholder)* | *“It needs to be made of a material which is not causing any itches or whatever and comfortable basically” (1. Participant)* | 7 |
|  | Environmental Consciousness | *“Rechargeable is more ecologically friendly isn’t it. I don’t know which is more ecologically friendly out of those two options”. (1. Participant)*  *“Eco-friendly is important to you?”*  *“I think so.”*  *“And to me” (2. Participant)*  *“I just think I mean, it's something that goes back to wearing things which are organic, things that are good for the environment. [Okay, Good for the environment.] So, this is an environmental question.” (3. Participant)*  *“You need to create something that your batteries can be changed because of that. Otherwise, people are spending a lot of money for a T-shirt after one year. They have to throw away and then they have to buy a new one. And your electronics are gonna go into landfill. You know all of that that we need to think about as tech.” (1. Stakeholder)* | *“Yes, the same I think it’s, most people won’t replace their shirt every three months so it seems wasteful.” (1. Stakeholder)*  *“It’s got to be recycled as much as possible we can’t throw things away unnecessarily.” (1. Participant)* | 6 |
|  | Cooperation by People | *“An experiment around physical activity, people who were told that they should be more active. What some of the people were doing would do, they were giving the walking stick to somebody else to take out with them or the monitor. So obviously, if somebody is really reluctant, someone's really reluctant to go out or do something, but they think that other people are monitoring them, then they can find a way of trying to make it look.” (1. Stakeholder)*  *“And the pedometer isn’t it, you just move your foot a few times and the, the buttons come, and it all seems to work very well for the phones as well.” (2. Stakeholder)*  *“I know some people that had pushed prams and they've had Fitbits and were annoyed because if you have your hands like that on a pram it doesn't monitor that you’re walking. So again, it's not conclusive, is it.” (3. Stakeholder)*  *“It's actually critical, is accuracy. I think you mentioned earlier that, you know, if people are given a walking stick with G.P.S. monitoring, they can give the walking stick to somebody else and wander off.” (4. Stakeholder)*  *“If people are willing to listen and it can be hard.” (5. Stakeholder)* | *“My mother is actually in a residential home. She started off with a mattress cover and a floor mat, but when she gets annoyed at the staff, she consciously hits it to make sure that the staff are required to come and see her. And they have given up with using them because she does it so often and they have to respond to her.” (1. Stakeholder)* | 6 |
|  | Aesthetics |  | *“It just looks like you are being imprisoned with a chain which is, unfortunately it’s not presented very well.” (1. Stakeholder)*  *“It does remind me of the original cars in America when everything was black, black, or black and this feels like it’s surgical. It doesn’t have a pretty pattern for example.” (1. Stakeholder)*  *“That one goes red, they wouldn’t like that. Now they know that I feel lonely, you know, because there is a stigma attached as well, so sweet colors, you know the meaning of them, but you don’t want to scare them.” (1. Participant)*  *“From well it needs to fit them and that is a big challenge and because you will have people of all sizes. But also, if they are still having some adventures, they want to look smart, even if they are in their seventies.” (1. Participant)* | 2 |
| Requirements for  Wearables and  Furniture | Compatibility with existing clothing | *“If you are wearing skirt or dress you are not going to want to wear socks, aren’t you?” (1. Participant)*  *“And if you got close fitting shoes, it’s possible that they wouldn’t, that I had to try the on, it is a bit difficult to tell, but I suspect those might be tricky.” (2. Participant)*  *“I don’t like wearing them anymore.” (3. Participant)*  *“If it was embedded in clothing, then you’d have, it would have to be in everything that you wore, you know, given that people wear quite a lot of clothes.” (1. Stakeholder)*  *“I've just marked up here and written a smart wearable would offer greater flexibility to a furniture retrofit or a clothing retrofit, because for the very reason you mentioned earlier, there are lots of different people who have lots of different clothing and different people have different types of furniture in their homes.” (2. Stakeholder)*  *“I just wonder how you get, you know, are we all going to walk around in orange clothes all at the same with our AI clothes on?... you know, this is a vintage dress my sister gave me. So, I don’t know the origins of where it came from, but you know, do I iron something in and then I've got it on there or how would I get that on or do I have to send my clothes off, which I might not be happy about.” (3. Stakeholder)*  *“Yeah.” (4. Stakeholder)* | *“Can you only buy the whole kit; you can’t buy individual parts?* *So, you couldn’t decide that actually you didn’t want the insole and the vest, but you did want the other things?... Well just because if somebody was refusing to wear the vest or the socks, then they might at least get the benefit of the other items.” (1. Participant)*  *A lot of people don’t wear watches anymore because they use their phones. I do, I prefer to have a watch but I know lots of older people who do not. (2. Participant)*  *People are particular about what they wear, you know, they spent a lifetime wearing Marks and Spencer or whatever and it definitely needs to be non-allergic stuff, definitely. Like cotton or whatever. And it needs to be market to standards, if it is approved, simplistic or whatever people will feel more easy. (3. Participant)*  *“So the insole would be in the shoe sort of, so like if you had three pairs of shoes, one to walk the dog and one to wander about in the house, I suppose you could have multiple insoles? You have multiple pairs of shoes right as well? [Oh, lots of pairs of shoes.] At least fourteen, you will have to give them an insole for that.” (1. Stakeholder)* | 11 |
|  | Compatibility with existing furniture | *“And furniture. Yeah, I guess it can be kind of personal to people like.” (1. Stakeholder)*  *“The furniture, I don’t think anyone would particularly want new furniture unless you really want to. You think it's very expensive as well you know.” (2. Stakeholder)*  *“No and sentimental value. I wouldn't change my furniture. Because some of the pieces are from my mum.” (3. Stakeholder)*  *“I don’t want to have to buy a new sofa.” (1. Participant)*  *“At this point in our life, we’d be unlikely to be replacing our furniture.” (2. Participant)*  *“I can't see people being able to afford to have new furniture.” (3. Participant)* |  | 6 |
|  | Seasonal | *“On a summer’s day you wouldn’t have it on the sleeve.” (1. Participant)*  *“If it was in the height of the summer and we were in a heatwave, I could see that becoming a different process. But it's but for majority of the time of year, that would be absolutely fine.” (2. Participant)* |  | 2 |
|  | Unisex | *“It would be a problem for men, presumably men, I mean we are all female.” (1. Participant)* |  | 1 |
|  | *Intuitive Wear* | *“I think the picture with this attached to your bra makes the most sense to me, because it is something you automatically put on and you wear under your clothing.” (1. Participant)* | *“I think the insole is better than the sock and the vest because the insole just literally goes in your shoe, not everybody has thirty pairs of shoes. But if you just have the insole in a couple of pairs of your most often worn shoes, I think that would be easier than remembering to put on the vest or the socks. I put the insole next after the other things.” (1. Participant)* | 2 |
|  | *Discretion* | *“I think people would ask you what it was. I think you would draw attention to it if it was on your wrist.” (1. Participant)*  *“If you had a highly visible, what if you had a high, I mean that would be counterproductive in some ways stigmatizing, it was a fabric strip that was highly visible cause it was a specific color and it was attached in an obvious place like around the wrist or the neck of the garment.” (1. Stakeholder)* |  | 2 |
|  | No detachable components on wearables | *“People will forget to remove stuff... Things will break because of that”. (1. Stakeholder)*  *“And also, it would have to be capable of being washed, if it's integral, So I’m thinking that something which was detachable or whatever. And the only thing I think about that is that when somebody if they start to get dementia, then they forget to attach things. So there's that thing about. I mean, obviously if you got dementia then if all your clothes for example have it inbuilt in, then you didn't have to think about it, that would be great. On the other hand, it would be quite a, quite a thing to do, wouldn't it.” (1. Stakeholder)* | *“It’s often with things like batteries to be removed, it’s not so difficult to remove it, it’s to put it back, so it would be easier for people to have the one where you don’t have to remove.” (1. Participant)* | 2 |
| Material Requirements | Lightweight | *“With wearables, you want to make it as seamless and lightweight and comfortable”. (1. Stakeholder)*  *“I don’t wear scarves very much anymore. Unless and necklaces and things, so if it had some sort of slightly stiff, I would find that tricky. [So, it would have to be very soft, is that correct?] It would have to be very soft and very light. And I might still want to throw it off.” (1. Participant)* |  | 2 |
|  | Soft | *“I don’t wear scarves very much anymore. Unless and necklaces and things, so if it had some sort of slightly stiff, I would find that tricky. [So, it would have to be very soft, is that correct?] It would have to be very soft and very light. And I might still want to throw it off.” (1. Participant)*  *“Because it's soft. It's got it looks like it's got a fairly solid base. It's warm. So, I'm going back to the warmth thing again, and it looks quite pleasant. I mean, I wouldn't mind wearing that. (2. Participant)* |  | 2 |
|  | Preference for Natural Materials | *“Any cotton would be fantastic, cause it’s breathable and I don’t overheat as much”. (1. Stakeholder)*  *No, not really. I mean. We have never reacted you know, like some people react to synthetics only generally wear cottons, but we are, I mean, personally I don't mind we didn’t mind anything. (2. Stakeholder)*  *“Well, that's why I'd be [fussy], because I like wearing natural fabrics.” (1. Participant)*  *“Well, I think in those are all [manmade] materials mostly, so I mean I, I'd like to use more natural products cause I'm in favour of natural” (2. Participant)* |  | 4 |
|  | Anti-Allergic | *“Some people react to synthetics only generally wear cottons,” (1. Stakeholder)* | *“And cotton I suppose because some people are allergic to, if I wear something artificial over a certain percentage, I get rashes all over the place.” (1. Participant)* | 2 |
|  | Non-Magnetic | *“I wondered if it would interact with your heart if you've got a pacemaker and if there's anything magnetic in it”. (1. Stakeholder)* |  | 1 |
|  | Wrinkle Free |  | *“Because some shirts nowadays, you know, you don’t really need to iron them” (1. Participant)* | 1 |
| Positioning  Requirements | Privacy | *“I didn't actually put one in the bed and it’s because basically for me, I thought to myself that was just a bit too private for me” (1. Stakeholder)*  *“They don't want to sense every time they go for a pee you know there's a sensor there. The same for us, of course.” (2. Stakeholder)*  *“Privacy areas and comfort I suppose.” (1. Participant)* |  | 3 |
|  | Usage in Daily Activities | *“I don’t think I’d mind it there, but it would be more vulnerable, when I am doing things like washing the dog and cleaning her and her jumping up. I think it would be vulnerable to damage almost in that place.” (1. Participant)*  *“Would you be limiting movement in as much, I am a very good girl and I do my exercises long and hard every morning, would I find it restricting.” (2. Participant)*  *“I’d be conscious of if I had a bag over my shoulder and had the strap going across.” (3. Participant)* | *“Then you would sort out the problem with the battery because the front of the battery will do. [Of the arm here?] Yes the front so then because they do sleep on the side.” (1. Participant)* | 4 |
| Washing Requirements | Washability of all system parts | *“If someone’s got incontinence problem, then they [are] more likely to have something that’s well washable, wipeable thing.” (1. Stakeholder)*  *“And also, you’d still have a bit left underneath that would not be as clean as it should be.” (1. Participant)* |  | 2 |
|  | Machine Washing | *“If you tell people that they have to hand wash something, then they're not going to like that.” (1. Stakeholder)*  *“I said I’d prefer the direct washing. A, simpler, but also if you got a length of Velcro on you it is kind of bulky. And stiff” (1. Participant)* | *“I only use one cycle on my washing machine and it’s going to destroy this. I use the hand washing cycle about once every five months. [Got it. And you are not willing to put a special cycle on for this shirt?] No.” (1. Participant)*  *“I would be the same.” (1. Stakeholder)*  *“I won’t hand wash.” (1. Stakeholder)*  *“As far as I am concerned, I wash a couple of times a week and I do use a delicate or a hand wash button on my machine.” (2. Participant)*  *“No, no I am happy to hand wash anything. I like doing hand washing. I hand wash a couple of times a week, so I hand wash my delicates, cashmere, bras, so I hand wash at the moment. I don’t mind removing stuff. I am happy to charge up phones, computers, I don’t mind any of that. So, I am happy with all of that.” (3. Participant)*  *“Yes, I think so because not everyone has got that kind of, not everybody enjoys hand washing, not everyone has got a washing machine that has that cycle.” (2. Stakeholder)*  *“So it could be, it depends on them and whether they suffer from arthritis but if they do, they might have a helper come in from time to time” (4. Participant)* | 7 (yes), 1 (not) |
|  | Life Span of Two Years | *“We have done a bit of a you know an estimate of how often things will get washed over a course of let's say two years”.* |  | 1 |
| Sensor Synchronization Device Requirements | Memory Support | *“What you want to do with this is not make people's lives harder. You're trying to make it easier, right? So, you need to think I would imagine you need to think how to make sure that you're not adding more things to think about to these people because you know, some of them might not remember things as easily.” (1. Stakeholder)* | *“It is much less likely that they forget about it if it is attached to their key or to their trousers.” (1. Participant)* | 2 |
|  | Prevention of Loss | *“I prefer it to be small. But I mean if it's too small… my mobile phone, I spend my life looking for it.” (1. Participant)* |  | 1 |
| Charging Requirements | Little Cognitive Strain | *“Having to remember how to do all of this stuff as well… I think if somebody else was doing it, that would be fine.” (1. Stakeholder)*  *“I assumed you were making it like a phone where you just plug... you don’t have to keep buying batteries like I hardly have batteries at home, so I prefer if I can charge something, and I imagine be the same less things to think about.” (2. Stakeholder)* | *Everything about this stuff, about the two items, the sock and the vest are the least popular I think around the table, isn’t likely to be effectively used and now we are talking about the complication of charging them and washing them. Some of these things are going to almost make themselves ineligible for the loneliness because of the complexity or the tiresome stuff. (1. Participant)*  *“Can they all be charged in the same place, so you have some kind of a charging mat, so that all your gadgets, all your separate bits, because they all go on the same thing?” (1. Stakeholder)*  *“That would be good… If there was a single place that you could throw the whole lot and they be charged in the morning.” (1. Participant)* | 4 |
|  | Fewer Plugs | *“We have a project with older people who also have learning disabilities. People don't necessarily have a lot of power, lot of plugs”. (1. Stakeholder)* |  | 1 |
|  | Notifications | *“If you then have your user your digital platform, that's where you can get notification and say ‘hey, it's low battery, you need to plug things like that’.”* | *“Obviously you need a little light to say I am charged because they might place it in“ (1. Participant)* | 2 |
|  | Accessibility | *“As you get older, fingers get a bit fiddley.” (1. Stakeholder)*  *“Your dexterity” (2. Stakeholder)* | *“I would have a comment about removable batteries and everything because I know of at least two people who have problems with hearing aids putting batteries in, and it depends how like fiddly it is. For people who don’t have the dexterity” (1. Participant)*  *“With old people, removal of the battery as well some of them can’t really grasp things, they have got arthritis and all sorts of things.” (2. Participant)* | 4 |
| Requirements for the Linked Intervention | Provide a Linked Intervention | *“I mean, I guess for me you know the big question is how can people be supported to deal differently with their loneliness. That feels to me the crucial bit. Because I don’t think it is difficult for people to identify that they are lonely. I don’t think it’s difficult for onlookers to perceive us as lonely… You know that’s the bit that needs the help. Until there is some way, that we can constructively imagine working with it, I don’t. I’m sorry if I’m being negative. But it feels to me that I then I can’t quite see how the value of the you know what the information is about.” (1. Participant)*  *“My overall feeling is that I am skeptical of its value and can imagine that it would be physically somewhat restrictive” (1. Participant)*  *“But what you’ve identified people are lonely, what then? Without being rude, what’s the point.” (2. Participant)*  *“Well, I haven’t got any family that would be useful. I don’t have children, but when you talk about the buddy system, do you mean that there would be a group of say a dozen people who sort of kind of got to know each other and you know there would be a limited group or would you be rung up by a stranger. Like alcoholics anonymous as you might say. But then if you knew tat group you could ring them yourself. [That’s what I’m saying.] At a time when you wanted to. I don’t see the value to measure this physiologically. [But I suppose in the long run that would be the point. Cause you have these people, and it would alleviate the problem.] But. It would be an awful lot of technology just to put a group of people together, wouldn’t it. Think of the money.” (3. Participant)*  *“I still remain deeply skeptical I have to say, but I would support the trial.” (3. Participant)*  *“I think I feel about that in a similar way.” (4. Participant)*  *“Because I don’t believe that my feelings of loneliness are reflected in physiological changes. And therefore, I don’t think there is anything useful for the sensor to sense.” (3. Participant)*  *“Yes, but if we are thinking of outcomes this is what appeals to me. I don’t want my family involved. I don’t want medical people” (4. Participant)*  *“You could argue that if you've if you’ve ident if a few people have been identified, then the sensor is most likely to be useful for people who have cognitive problems, who are not or communication*  *problems who are not able to act on the issues that they may have. So therefore, they may be unable to really remember whether they've done something social or not that would potentially be say someone with a level of dementia who can't remember if someone has visited them or can't remember if they've been out or what they've been doing, then you could say a sensor is useful for them because it's giving information that they're unable to really accurately convey.” (1. Stakeholder)* | *“I don’t see the point I am sorry if it sounds rude. I just don’t see the point of saying ‘oh yes I didn’t know I was lonely until I used this’.” (1. Participant”*  *“Well yes sorry, to me I said before to [RES2] was it, sorry, I am not sure what help I will be for this research. If I know I am isolated or lonely, why do I need that? I don’t need that to tell me. (1. Participant)*  *“It is useful to use the system as an older person that experiences loneliness. Yes, it depends on what you do about it. I would say again this can be used intuitively by older people, yes. The system is useful to reduce loneliness for older people, not in itself. It depends on what is done because you are just monitoring. But if you started doing for example the weekly meetings and you get them involved and so on, then yes definitely.” (2. Participant)*  *“Well, I am implying that loneliness is a condition that, with cancer it’s a medical problem that can have various treatments, the question that I think you are asking is what are the treatments?” (3. Participant)*  *“I think the potential of this is very good because when my father started with dementia and he was four hundred miles away from the nearest family member, this would have been fantastically helpful to me. If I could have seen that he hadn’t got out of bed, that he hadn’t opened the fridge, that he had been sitting on the sofa all day, this would have been really, really helpful. Instead, I had to rely on phoning him up two or three times a day and if he didn’t answer I had to phone a neighbor and the neighbor would go over to see if he was okay. So, for a short time until we got some other solutions, this would have been great, I would have paid anything for this.” (4. Participant)*  *“For dementia I think it’s wonderful.” (3. Participant)* | 9 |
|  | Personalization | *“I think it should be personalized because if it is just coming out with, ‘have you been to a knitting group’, [swearword]” (1. Stakeholder)*  *“It should be perhaps localized a little bit because people often I go to something in the local area because it's easier to travel to and things like that and you want to take away as many barriers as you can. Or people will travel for the right thing, but often they need someone to go” (1. Stakeholder)*  *“All of your interests. Something that really interests you and you will make the effort.” (1. Stakeholder)*  *“Or do you reach out to other people with different language, you know, the same sort of languages or same sort of cultural view, I think. I think because then often people, if you with it might be that you feel as though you're not with people you relate to. So then if they could put you in touch with other people that you relate to.” (1. Stakeholder)*  *“Or could you if each person fed into the system originally what it was that made them feel less lonely. So, they said, well, what makes me feel less lonely is talking to my neighbor Bob or going out to the group I know exists, you know, every Tuesday. So, if they could do that on a personal basis, then the message could be, have you had a chat with” (2. Stakeholder)*  *“It should be relevant. Yeah, it should be personalized.” (3. Stakeholder)*  *“Make it hybrid. If people are willing to have sensors which are in the furniture. But if people are*  *willing to wear sensors around their neck, let's make sensors hybrid. If I want to wear something*  *around my neck as a sensor every morning…Like pendant alarms. Let's have a hybrid sensor. And those of us who might not remember to put it*  *on or forget it in the bathroom or something, then let them have the choice of having sensors*  *embedded. So, let's have hybrid sensors.” (3. Stakeholder)* | *“Right. I was interested to see choir at the top of one list that you had because that is a very good example of me. I am in a choir, a very successful touring all over the world choir. I have lost all interest in it and any excuse to not go to rehearsal I will pick up on it. So, it’s all very well saying oh you would like this. I sing so yes you would like this. But I don’t want. So that’s my personal thing.” (1. Participant)*  *“I think it’s drilling down to what motivates people individually.” (1. Stakeholder)*  *“Absolutely yes because everyone’s different.” (1. Participant)* | 5 |
|  | Avoid Burden to Family Members | *“I don’t want it to be family because I think I mean I’ve got this memory of my grandmother guilt tripping people all the time. And I think this would be a very formal way of guilt tripping. And you know, they’ve got their own lives.”*  *(1. Participant)*  *That's not a good thing, but it's a reality that they could just, or their situation is such that they can't manage, they're overwhelmed themselves. Cause actually sometimes the people that help us get overwhelmed and can't. (2. Participant)* |  | 2 |
|  | Infrastructure | *“And do we have the infrastructure to support it?” (1. Stakeholder)*  *“You felt you were having a heart attack and you phoned nine, nine, nine, it still might take six hours. So, I suppose that's not that this doesn't change that, does it. That’s an extra thing.” (2. Stakeholder)*  *“But the other thing is what about the situational people, so people that are caring for somebody. So, it might be someone that you know is going to, you know, you've got a progressive illness, say your life’s pretty hellish because she can't really leave that person until the inevitable, which is what happened to my dad. He was a carer for my mum for many years and then the inevitable has happened and so his loneliness was because he was tied in and couldn't do things, cause she wouldn’t let him do things. And now his loneliness is different, but he can now do something about it because he's not talked to anyone who can do lots of things. So, his loneliness has changed. Or if you're at home with young children, it can be a really lonely place on your own if you, you know, single parents or whatever your situation is. But you know that once the children go to school, your time is more your own to be able to do a bit more stuff. So sometimes it's a situation so, you know, it's chronic. So, you need to kind of find ways to manage that. So, getting is a way of managing a situation that's not gonna change because it can't for a while. Or is it ‘Here's some interventions you're going to feel better, and off you go’” (2. Stakeholder)*  *“Is a phone call from my daughter gonna be enough? If she's busy, she's only got like 2 minutes to talk on the phone. Is that gonna do anything?” (3. Stakeholder)* | *“A while ago my daughter was concerned with my loneliness and stuff, and I spoke to the GP and the GP had somebody phone me. I can’t remember what the title of this person was. And she spent half an hour on the phone to me and said she would get back to me the following week.* *I think it was about six months later she got back to me, and I am not joking… So, your GP first of all has to, the way things, the NHS is, they have got far more important things to deal with than that, so would it bring any results?” (1. Participant)* | 4 |
|  | Motivation |  | *“I suppose thinking of previous roles I’ve had in housing, these types of roles, the people that I would meet wouldn’t necessarily be, you know. I appreciate that it’s about loneliness can affect people in different ways and we know that it’s a high-risk factor for lots of heart conditions. I am just wondering whether people, depending on what levels we might be at, like their loneliness or their health, whether or not they would be motivated to or able to do these kinds of things because their mental health potentially…I am thinking they won’t necessarily even wash their clothes.” (1. Stakeholder)*  *“[So, you don’t think they would be willing to do this washing?] Exactly. It’s the maintenance and the things they need to do to keep, you know, the things they will need to do daily like charging and washing and things like that.” (1. Stakeholder)*  *“For people who are lonely or who are isolated, it’s generalized again but they are not necessarily, they are in that situation because they potentially aren’t the type of person … or there aren’t the opportunities to go and do something. Actually, stepping through the door of some kind of group activity for some people they just wouldn’t do it.” (1. Stakeholder)*  *“Because you like, you don’t go to the choir but perhaps there is something else that could, something of an intervention that could help you regain that motivation. And that’s perhaps it’s the drilling down in to get to know people and what motivates and what people’s passions are” (1. Stakeholder)*  *“A suggestion of events and things? Like I said before that is a whole different subject, it’s all very well to suggest it, you have got to want to do it. You have got to feel motivated to do it. It’s just much easier to sit with your feet up and you can’t be bothered to go out, then what’s the point of suggestions?” (1. Participant)*  *“I can only say for me, no I wouldn’t be motivated. Sometimes when I have forced myself to do things because I think it will do me good, I have regretted it. [Oh really?] I wish I could have just stayed at home.” (1. Participant)*  *“You need to drive people out of their place because they will tend to hide in the winter months especially in places where they don’t feel secure, you know for going out. Whether it is a combination of say electrical scooters on the pavements, it is enough that you are bumped into, break the hip, you are dead in six months, you never recover at a certain age, yes, people are scared, yes. So there needs to be centered around the GP hub, library or they need to work in conjunction, they are already pooled together into social services. And the people they refer to them they will know that they are coming from someone who is suffering from loneliness as well. There will be activities already around on an ongoing basis for these kinds of people and facilitated, there will be some soft spiritual care there so that they are ready to pick up the signals and keep the conversation with one or the other.” (2. Participant)* | 3 |
| Installation and  Maintenance Requirements | Identification | *“I think identification is really important as well.” (1. Stakeholder)*  *“You need to know whether they’re likely to be lonely in the first place.” (2. Stakeholder)*  *“I think sometimes it's people who specific help bit of the health service, where people have gone*  *regularly, so say they've got a chronic health problem. So, in a good example, my mother was told by*  *the GP she didn't definitely didn’t have dementia; he did a test. But then six months later the*  *rheumatology department where she'd been going for years, the nurse there said to her, I think*  *you've got a problem with your memory. And she only did that. She knew because she'd seen my mum for years and she noticed a difference. So, in a sense it's got to be someone who has sufficient knowledge of you and who you’re gonna be open with. And it could be a range of people really couldn't they. It could be a charity, a local charity. Somewhere you go, you might go to a local*  *club or something.” (2. Stakeholder)*  *“Because it is stigmatizing saying you're lonely can be really difficult. So, if I said to some people, oh I feel lonely, they’d be like ‘Oh, don’t be ridiculous, you know, you’re always busy doing something’, ‘Yeah, but actually I do that because I feel lonely’. And then some of the time it doesn't kind of work, do you know what I mean, or do we just say everyone that moves into this extra care housing has this as part of it. And then it's not your choice. You just move into that house and it's part of your normal living” (3. Stakeholder)*  *“You know what I think is good as well: delivery services, you know, people that that can't go out of*  *the house, and they have the supermarket delivery unless they get in the shopping delivered.” (3. Stakeholder)*  *“It could be worthwhile considering getting Asda or Tesco or Sainsbury’s to champion such a service*  *by providing them with the appropriate training for their drivers, who are actually meeting many*  *people face to face, many of whom may well be lonely. As you've said.” (4. Stakeholder)* | *“Over sixty-five yes, I mean there are thousands and thousands of people living in our housing alone. So there is no way, I don’t think, that we could kind of cost that….I think it would be more likely that this kind of thing could be incorporated into our housing scheme so it’s something that’s kind of offered to people as they move in other than people who already live there… if the housing provider was to pay for this, like I said because we are not for profit we have to recuperate that cost somewhere. [And where would that cost come from?] From people’s rents. [Right, okay.] And so therefore that’s why it would work better in a scheme of flats or apartments because they pay a service charge towards that, whereas people in our community you couldn’t, you impose a charge on somebody that the neighbor hasn’t got, you see.” (1. Stakeholder)* | 5 |
|  | Consent | *“I'd like to know. My life, my choice. I am compos mentis. I want to be part of that choice. My children for me would be part of the people I would love to have part of it.” (1. Participant)*  *“First thing needed is consent.” (1. Stakeholder)*  *“Correct. That is where social services come in.” (2. Stakeholder)*  *“The person whose data people are accessing, they need to give people permission and they might not want to share certain things with the carer, they might not want to share with the family”. (3. Stakeholder)* |  | 4 |
|  | Easy Installation | *“The system will arrive in a way that's easy to use a plug and play kind of thing, so it will have maybe a booklet with instructions, so someone could use it themselves”. (1. Stakeholder)* |  | 1 |
|  | Instruction for Carers | *“Carers are never the same. They can unplug things; they forget to charge things because they don't know that they need to charge something… if a carer is then given something that says, ‘OK in this house we have this installed and this is what it does, and part of your job is to also make sure that this is charged and this works.’” (1. Stakeholder)* | *“Well, someone else might need to take care of it, you know. Beyond a certain age someone else will probably help them out with the chores and then again, they would need the instructions.” (1. Participant)* | 2 |
|  | Continuous Support Provision | *“The option to call as a customer services if they have issues.”* |  | 1 |
